# The CREB-binding protein inhibitor ICG-001: a promising therapeutic strategy in sporadic meningioma with *NF2* mutations

**DOI:** 10.1093/noajnl/vdz055

**Published:** 2020-02-22

**Authors:** Jiaojiao Deng, Lingyang Hua, Tao Han, Mi Tian, Daijun Wang, Hailiang Tang, Shuchen Sun, Hong Chen, Haixia Cheng, Tao Zhang, Qing Xie, Lixin Wan, Hongda Zhu, Ye Gong

**Affiliations:** 1 Department of Neurosurgery, Huashan Hospital, Shanghai Medical College, Fudan University, Shanghai, China; 2 Department of Molecular Oncology, H. Lee Moffitt Cancer Center and Research Institute, Tampa, Florida; 3 Department of Critical Care Medicine, Huashan Hospital, Fudan University, Shanghai, China; 4 Department of Neuropathology, Huashan Hospital, Fudan University, Shanghai, China; 5 Department of Laboratory Medicine, Huashan Hospital, Fudan University, Shanghai, China

**Keywords:** β-catenin, FOXM1, ICG-001, Merlin protein, *NF2*-mutant meningioma

## Abstract

**Background:**

Meningiomas with Neurofibromin 2 gene mutations (*NF2*-mutant meningiomas) account for ~40% of the sporadic meningiomas. However, there is still no effective drug treatment for the disease.

**Methods:**

Expression profile of Merlin protein was explored through immunohistochemistry in a meningioma patient cohort (*n* = 346). A 20-agent library covering a wide range of meningioma relevant targets was tested using meningioma cell lines IOMM-Lee (*NF2* wildtype) and CH157-MN (*NF2* deficient). Therapeutic effects and biological mechanisms of the identified compound, ICG-001, in *NF2*-mutant meningiomas were further characterized in vitro and in patient-derived xenograft (PDX) models.

**Results:**

Low Merlin expression was associated with meningioma proliferation and poor clinical outcomes in a large patient series. ICG-001, a cAMP-responsive element binding (CREB)-binding protein (CBP) inhibitor, selectively suppressed tumor growth of cells with low Merlin expression. Besides, ICG-001 mediated CH157-MN and IOMM-Lee growth inhibition primarily through robust induction of the G1 cell-cycle arrest. Treatment with ICG-001 alone significantly reduced the growth of *NF2*-mutant xenografts in mice, as well. We also provide further evidence that ICG-001 inhibits proliferation of *NF2*-mutant meningioma cells at least partly through attenuating the FOXM1-mediated Wnt/β-catenin signaling.

**Conclusions:**

This study highlights the importance of ligand-mediated Wnt/β-catenin signaling as well as its drugable potency in *NF2*-mutant meningioma.

**Key Points**

- Low Merlin expression was associated with meningioma proliferation and poor clinical outcomes.
- Low Merlin expression sensitizes meningiomas to ICG-001 treatment, at least partly, by attenuating the FOXM1-mediated Wnt/β-catenin signaling.

**Importance of the Study**

Some ongoing clinical trials are looking for new methods to treat refractory meningiomas in order to obtain better prognosis. However, there are currently no FDA-approved drug therapies for the treatment of *NF2*-mutant meningiomas. We conducted the largest cohort study showing the expression and clinical significance of the Merlin protein, the *NF2* gene translated product, in meningioma patients. Based on drug testing, the Wnt/β-catenin pathway, which is blocked by ICG-001, is important for the growth and survival of *NF2*-mutant meningiomas. Our study highlights the importance of ligand-mediated Wnt/β-catenin signaling as well as its targetable potency in *NF2*-mutant meningiomas.

Meningiomas are the most common primary intracranial tumors in adults and ~80% are classified by the World Health Organization as WHO grade I lesions.^1,2^ The WHO grade II–III group comprises ~20% of all meningiomas characterized by aggressive biology with increased proliferation and a strong tendency to recur, resulting in substantial morbidity and increased mortality.^2^ Although most benign meningiomas can be treated with surgical resection, high-grade meningiomas that exhausted all surgical or radiotherapeutic options still need novel therapies to improve outcomes.^3^ Recent high-throughput studies have defined novel driver mutations that strongly associate with clinicopathologic features.^4,5^ Loss of the Neurofibromin 2 (*NF2*) gene, located on chromosome 22, is one of the main genomic alterations found in high-grade meningiomas.^6^ Splice site, nonsense, and frameshift mutations are the most common disruptors of *NF2*, resulting in a truncated and nonfunctional protein.^6^ Moreover, *NF2*-mutant cases often present multiple and malignant tendency, inducing severe neurologic morbidity and mortality.^2,6^ The lack of effective systemic therapy for meningiomas represents a significant challenge in the clinical management of patients with *NF2* mutations.

Merlin, the *NF2* gene translated product, is a cytoskeletal protein that provides a functional link between the cell membrane and the actin cytoskeleton.^7^ A number of studies demonstrated that Merlin is a versatile tumor suppressor that can inhibit cancer cell proliferation and motility by modulating a wide range of signaling pathways. For instance, active Merlin suppresses Rac-PAK signaling,^8^ restrains activation of mTORC1 independently of Akt,^9^ inhibits PI3K-Akt and FAK-Src signaling,^10,11^ and negatively regulates the EGFR-Ras-ERK pathway.^12^ Merlin has also been reported regulating the Hippo tumor-suppressor pathway in meningiomas.^13^ Besides, the loss of Merlin results in aberrant activation of the Wnt/β-catenin signaling in brain tumors.^14^ Forkhead box M1 (FOXM1) is a key mediator of the Wnt/β-catenin signaling, which binds to β-catenin, thus enhancing β-catenin nuclear localization and transcriptional activity.^15,16^ Recently, FOXM1/Wnt signaling was found to be associated with mitotic gene expression in aggressive meningioma.^17^ Thus, we are interested in potential functional connections between these two intensively studied oncogenic pathways.

Prospective clinical trials are evaluating novel drugs based on the further understanding of the pathobiology of treatment-resistant meningiomas. The ongoing preclinical studies also presented several targets for systemic therapies, seeking treatments for neurofibromatosis 2–related meningioma (www.synapse.org/SynodosNF2).^18^ Yet despite these advances, targetable pathways associated with poor meningioma outcomes remain elusive. A number of drugs including hydroxycarbamide, interferon-alfa, megestrol acetate, medroxyprogesterone acetate, octreotide, pasireotide (long-acting release), trabectedin, imatinib, erlotinib, gefitinib, vatalanib, sunitinib, and bevacizumab, as well as cyclophosphamide-doxorubicin-vincristine chemotherapy, have been evaluated.^19,20^ Unfortunately, most of the small and uncontrolled studies and case series suggest that systemic therapies have only minimal activity against aggressive meningiomas.^21–24^ The high failure rate of preclinical agents in clinical trials clearly demonstrates that even when targetable genomic alterations are discovered, patients do not always respond to therapy.

Strategies to confirm therapeutic efficacy or identify additional options would be beneficial to both clinicians and patients. To address this need, we screened 10 patient-derived primary tumor cell lines and two high-grade meningioma cell lines with a panel of agents under clinical and preclinical investigation. A focused drug screen showed that *NF2*-mutant meningioma cells are especially sensitive to ICG-001, a cAMP-response elemen (CREB)-binding protein inhibitor, both in vitro and in vivo. The effect of ICG-001 is at least partly through attenuating the FOXM1-mediated Wnt/β-catenin signaling. Our results thus advocate ICG-001 as a promising agent for further preclinical evaluation in *NF2*-mutant meningiomas.

## Materials and Methods

### Ethics Statement

We recruited 355 adults with primary meningioma who underwent surgery from 2008 to 2017 in the Department of Neurosurgery, Huashan Hospital of Fudan University, Shanghai, China (Supplementary Tables S1 and S2). Four normal arachnoidal tissues collected from brain trauma patients were set as control. All patients gave their written informed consent and were enrolled in institutional protocols (#KY2012-019) approved by the Human Subjects Institutional Review Board.

### Tissue Microarray and Immunohistochemistry

Tumor specimens for drug testing were classified as wildtype or associated with the *NF2* mutation based on diagnoses from referring pathologists. Patients with a family history of neurofibromatosis type 2-related meningioma history were not recruited for this study. The tissue microarray (TMA) constructions were sectioned (5 μm) according to standard protocols for hematoxylin and eosin (HE) staining. The TMA immunohistochemical analysis was conducted with anti-Merlin, anti-FOXM1, β-catenin, and anti-Ki-67 (CST; 9449) antibodies as described previously.^25^ The staining TMAs were scanned (Pannoramic MIDI, 3D HISTECH) and analyzed by Quant center software. Histoscore (H-SCORE) was calculated according to the following formula：H-SCORE = ∑（PI × I）=（percentage of cells of weak intensity × 1) + (percentage of cells of moderate intensity × 2) + percentage of cells of strong intensity × 3).^26^

### Cell Culture

CH157-MN and IOMM-Lee meningioma cell lines were a courtesy of Prof. Wan’s lab (H. Lee Moffitt Cancer Center, Tampa, FL) and cultured in DMEM/F12 medium (Gibco, 11320033) with 10% Fetal Bovine Serum (FBS, Sigma, F8192). Primary meningioma cells were separated from high-grade meningioma patient tissue and cultured in DMEM/F12 medium containing 2% B27, 1% N2, bFGF, and EGF (20 ng/ml each) supplement (Invitrogen). Spheres from three independent experiments (day 7) were plated in standard culture medium with 10% FBS to allow cells to adhere. Tumor cells were identified by Vimentin (Abcam, ab92547, 1:500 dilution) and epithelial membrane antigen (EMA) staining (Abcam, ab156947, 1:100 dilution) before drug screening.

### Drug Test

CH157-MN, IOMM-Lee, and primary cells were cultured for 12 hours before the compounds were added. To perform dose–response screening, agents were tested in a nine-concentration semilog serial dilution ranging from 0 to 20 µM with eight replicates for each concentration. ATP levels were analyzed following compound treatment for 72 hours using the Cell Counting Kit 8 assay (CCK8, Abcam, ab228554) according to the manufacturer’s recommendations. The percentage of viable cells for each agent was calculated by normalizing the raw data to the mean of DMSO control wells (100% viability). The absolute half-maximum effective concentration IC_50_ and the maximum effect for each compound were calculated in the dose–response screens.

### RNA-Sequencing and Data Analysis

RNA was extracted from three biologic replicates of CH157-MN and IOMM-Lee cells 8 and 24 hours after treatment with 5 μM ICG-001 or DMSO. The RNA quality and integrity were analyzed by Qubit 2.0 (Life Technologies, USA) and Bioanalyzer 2100 (Agilent, Germany). The sequencing library was qualified by Qubit 2.0 (Life technologies, USA) and Bioanalyzer 2100 (Agilent, Germany) and then sequenced on Illumina Hiseq X with 2 × 150 bp paired-end sequencing, which were controlled by Hiseq Control Software (HCS). Candidate genes were filtered to a minimum of 2× fold changes and FDR-corrected *P*-value < .05. For functional analysis, gene ontology enrichment was tested accounting for gene length via R-package goseq. GO-annotation analyses were performed using DAVID Bioinformatics Resources 6.8 (Frederick, USA).^27,28^ RNA-seq data can be accessed from the BPbrowse database (http://122.112.248.194/GMOD/BP_yun/?data=sample_data/json/BPdata/Project_1711SZSC03B/data).

### Cell-Cycle Analysis and EdU Assay

For cell-cycle analysis, cells were treated for 2 hours prior analyses. Cells were washed in PBS and then trypsinized. All the material was collected, combined, centrifuged at 15,000 rpm for 5 minutes, and washed with PBS. Pellets were resuspended in 500 µl binding buffer and stained with propridium iodide (PI). Cell-cycle analysis was performed using Summit 5.2 software. Following designated treatment, cells were pulsed with 5-ethynyl-2-deoxyuridine (Click-iT Plus EdU Alexa-647 Imaging Kit, Life Technologies), for 2 hours before fixation in 4% paraformaldehyde and subsequent EdU detection as per the manufacturer’s protocol. Coverslips were mounted on slides and imaged using a confocal microscope. Quantification of EdU+ tumor cells was carried out using the cell counter plugin from the Image J software (d 1.47 version). The percentage of EdU+ for each field of view captured was recorded and analyzed.

### Immunoblot and Immunoprecipitation

Immunoblotting analysis was performed as described previously^25^ using antibodies specific for β-catenin [Cell Signaling Technology (CST); 8480], CBP (CST; 7389), Merlin (Santa Cruz; sc-331), FOXM1 (Santa Cruz; sc-376471), p21 (CST; 2947), survivin (CST; 2808), and β-actin (CST; 4970). For immunoprecipitations, cell lysates and indicated antibodies (1 µg) were subjected to overnight rotation at 4°C. Protein A-Sepharose beads (40 µL, GE Healthcare) were added for 3 hours, followed by centrifugation, washed four times, and boiled with loading buffer for immunoblotting analysis.

### In Vivo Xenograft Studies

All animal studies were conducted in accordance with the guidelines as published in the Guide for the Care and Use of Laboratory Animals.^29^ Patient tumor material was collected in culture medium and kept on wet ice for engraftment within 12 hours after resection. The patient-derived xenograft model was established by direct implantation of 20–30 mg of surgically resected tumor tissue into the flank region of female BALB/c nude mice (6–8 wk old; HFK Bioscience Co., Ltd, Beijing) followed by a second round of serial propagation in nude mice. Intraperitoneal drug injections were administered once the tumor volume reached about 30 mm^3^: ICG-001 (10 mg/kg, once a week; *n* = 5) and vehicle alone (20% PEG300, 5% solutol, 3.75% dextrose, 1% DMSO in PBS; *n* = 5). The treatment was extended for a total of 8 weeks. Tumor size and animal body weight were measured on designated days.

### RNA Interference Studies

The plasmid pcDNA3.0-Flag-tagged Merlin was the full-length Merlin isoform I and was obtained from Addgene. Mutated Merlin-S518A was generated via overlapping PCR and inserted into pcDNA3.0 (pM-S518A) and pcDNA3.0-HA (HA-M-S518A) vectors. Targeting FOXM1 (siFOXM1) consisted of a pool of three target-specific 20- to 25-nt siRNAs designed to knockdown FOXM1 expression and was obtained from Santa Cruz Biotechnology (sc-37615; sc-43769; sc-270048). After transfection, cell proliferation was measured with the CCK8 assay according to the manufacturer’s instructions. Cells were collected on day 3 to check for knockdown efficiency.

### Statistical Analysis

All statistical comparisons between two groups were performed using GraphPad Prism software 6.0 and a two-tailed unpaired *t* test, unless otherwise noted in the figure legend. Survival curves were generated using the Kaplan–Meier method; differences were assessed by log-rank tests. Data were considered to be significant when *P* < .05.

## Results

### Low Merlin Expression Is Associated With Meningioma Proliferation and Poor Clinical Outcomes

**Fig. 1:**
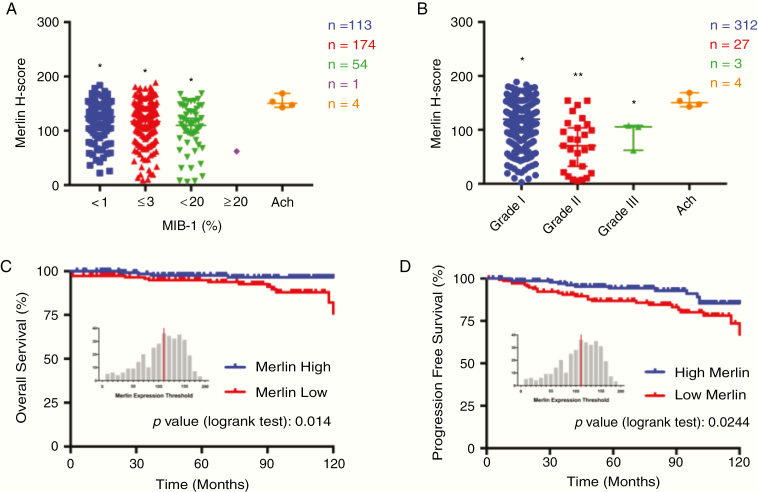
Low Merlin expression is associated with meningioma proliferation and poor clinical outcomes. (A) Merlin expression in meningioma patients with different mitotic activity. *N* = 346, *P < .05, ANOVA. (B) Decreased Merlin expression was significantly correlated with the increased pathological grade. *N* = 346, *P < .05, ANOVA. (C and D) Decreased Merlin is associated with worse outcome in patients with meningioma. Patients were split by median expression of Merlin over the entire meningioma data set (313 patients). Patients with lower than median expression of Merlin (*n* = 160) are denoted in red, and those with higher than median expression of Merlin (n=153) are in blue.

A total of 346 patients with primary meningioma (237 females, 109 males) and a mean age of 52.29 ± 15.7 years (range 19–86 y) were included in this study. Thirty-three patients (9.54%) did not attend follow-ups and were excluded from the final analysis. For the remaining 313 patients, the median follow-up was 76.5 months (range 1–142 mo). Clinicopathological characteristics of patients with low or high Merlin expression are summarized in Supplementary Figure S1 and Supplementary Table S1. Merlin protein expression was statistically significantly different (*P* < .05) between groups of different mitotic activity in meningioma (Fig. 1A), as defined by MIB-1 (Ki-67) labeling index (Mitotic index ≥ 4 mitoses/10 HPF and Mitotic index ≥ 20 mitoses/10 HPF) according to the WHO 2016 grading criteria for meningioma.^2^ We also performed differential expression analysis of Merlin on the basis of the meningioma grade, which classically define aggressive meningiomas. Our results revealed that the decreased Merlin expression was significantly correlated with the increased pathological grade (*P* < .001) (Fig. 1B). No significant differences were observed for patient age, gender, tumor location, and extent of resection between these two groups. Clinical variable analyses revealed that low Merlin protein expression was also significantly associated with decreased progression-free survival (PFS, *P* = .0244) and decreased overall survival (OS, *P* = .014) (Fig. 1C and 1D).

### Focused Drug Testing Identifies ICG-001 as a Potential *NF2*-Associated Meningioma Therapeutics

**Fig. 2:**
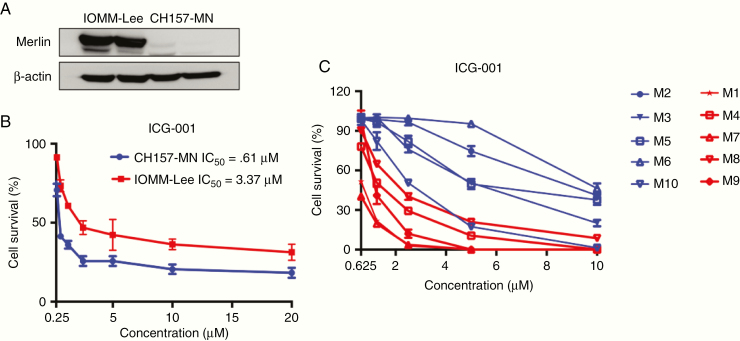
ICG-001 selectively inhibits proliferation of meningioma cell lines in vitro. (A) Western blot analysis of Merlin levels in IOMM-Lee and CH157-MN cells. (B) Dose response of IOMM-Lee and CH157-MN cells treated with ICG-001 for 72 hours (*n* = 8). (C) Dose response of 10 patient-derived meningioma cells with different Merlin expression levels treated with ICG-001 for 72 hours (*n* = 8).

We performed a drug screening using human malignant meningioma cell lines IOMM-Lee (*NF2* wildtype), CH157-MN (*NF2*-deficient) (Fig. 2A), and a 20 agent-library encompassing both targeted agents and cytotoxic chemotherapeutics. These agents target meningioma pathological relevant molecules and signaling pathways, including RTKs, Raf, PI3K, mTOR, MEK, WNT/β-catenin, and Hedgehog/Smoothened (Supplementary Table S3). We assessed tumor cell viability in response to 72 hours of treatment at four different concentrations ranging from 0 to 20 µM. In the initial screening, we found ICG-001, a cAMP-response element (CREB)-binding protein inhibitor, that selectively inhibited proliferation of CH157-MN cell lines in vitro (Fig. 2B). Besides, several agents previously reported to inhibit meningioma involved signaling such as Everolimus, Vemurafenib, and Trametinib reduced CH157-MN and IOMM-lee cells survival at tested doses (data not shown).

To investigate the potential of ICG-001 for treating *NF2*-mutant meningiomas, we performed another testing with ICG-001 in ten high-grade patient-derived cell lines (Fig. 2C). Cell viability was assessed in the 10 primary cultures at passages 2–4, and for all of these cases, >90% of cells were viable. Primary meningioma cells were characterized using cytology and histology as described previously^25^ (Supplementary Figure S2). Specifically, ICG-001 was predominantly effective on cells lacking Merlin protein expression (average IC_50_ = 1.17 μM, range from 0.696 to 2.005 μM), whereas among the less responding cell lines (average IC_50_ = 6.29 μM, range from 2.507 to 9.764 μM), most of them expressed high Merlin levels (Fig. 2C).

### ICG-001 Inhibits the Expression of Genes Involved in Cell Cycle and Induces G1 Arrest In Vitro

**Fig. 3:**
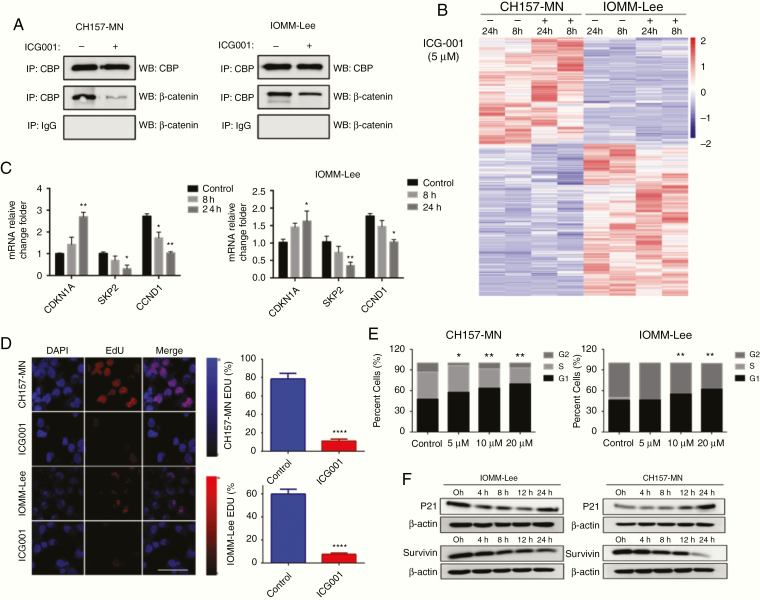
ICG-001 disrupts the interaction between β-catenin and CBP and robustly induces G1 arrest in meningioma cells in vitro. (A) The interaction between CBP and β-catenin was disrupted in CH157-MN and IOMM-Lee cells by 5 μM ICG-001 treatment for 12 hours as measured with coimmunoprecipitation. (B) A heat map of a core set in CH157-MN and IOMM-Lee cells treated with 5 μM ICG-001 at 8 and 24 hours, respectively. (C) mRNA levels for genes link to cell cycle after treatment with ICG-001. *P* values are derived from Wald test statistics in the DESeq2 package. (D) EdU assay shows CH157-MN and IOMM-Lee cells treated with ICG-001 at 5 μM resulted in a significantly decrease in proliferation (*n* = 3, *P* < .001). (E) ICG-001 treatment robustly induced G1 arrest in CH157-MN and IOMM-Lee cells (*n* = 3 for each dose, *P* values depict differences as dose-dependent manner). (F) Western blot revealed corresponding increased p21 and decreased survivin protein expression within 4 to 24 hours following ICG-001 treatment.

As previously shown for other Wnt-driven tumors,^30,31^ ICG-001 disrupted the interaction between CBP and β-catenin in CH157-MN and IOMM-Lee cells at different levels when measured by coimmunoprecipitation (Fig. 3A). The CBP is a coactivator of Wnt/β-catenin-mediated transcription; we thus performed RNA sequencing of CH157-MN and IOMM-Lee cells to investigate the effects of ICG-001 treatment on global gene expression. In total, 646 transcripts of CH157-MN (298 upregulated and 248 downregulated) and 651 transcripts of IOMM-Lee (389 upregulated and 262 downregulated) showed altered expression after ICG-001 treatment for 24 hours, respectively. ICG-001 treatment altered the expression of numerous genes linked to the cell cycle (i.e., *CDKN1A*, *SKP2*, *CCND1*, and *CDC2A*) in RNA-seq microarray analysis (Fig. 3B and 3C). Top gene ontology (GO) terms for genes associated with ICG-001 at the 8-hour time point revealed a series of molecular events involved in cell cycle G1/S phase transition (*p* valve: 2.15E-07, FDR: 3.60E-04) and G1/S transition of the mitotic cell cycle (*p* valve: 3.29E-07, FDR: 3.64E-04) biological process in CH157-MN (Supplementary Table S4). However, enriched genes of IOMM-Lee at the same time point were involved in negative regulation of protein phosphorylation (*p* valve: 1.89E-11, FDR: 8.25E-08) and cellular response to oxidative stress (*p* valve: 1.66E-06, FDR: 8.10E-04), which means IOMM-Lee resisted damage, whereas CH157-MN was more vulnerable to ICG-001 treatment (Supplementary Table S4).

Both CH157-MN and IOMM-Lee cells treated with ICG-001 resulted in a significantly decrease in proliferation as evidenced by reduced EdU incorporation (Fig. 3D). The exposure of CH157-MN and IOMM-Lee cells to 5 μM ICG-001 for 8 hours both induced a significant G1/S accumulation (*P* < .05, vehicle vs. 5 μM ICG-001) (Fig. 3E). Noticeably, 5 μM ICG-001 treatment robustly induced G1 arrest in CH157-MN cells, whereas no significant result was observed at the same dosage in IOMM-Lee cells. Further detailed time-course analyses of protein expression showed corresponding increase in p21 protein expression and decrease in survivin protein expression within 4 to 24 hours after the ICG-001 treatment (Fig. 5F).

### ICG-001 Reduces *NF2-Mutant* Meningioma Patient–Derived Xenografts Growth

**Fig. 4:**
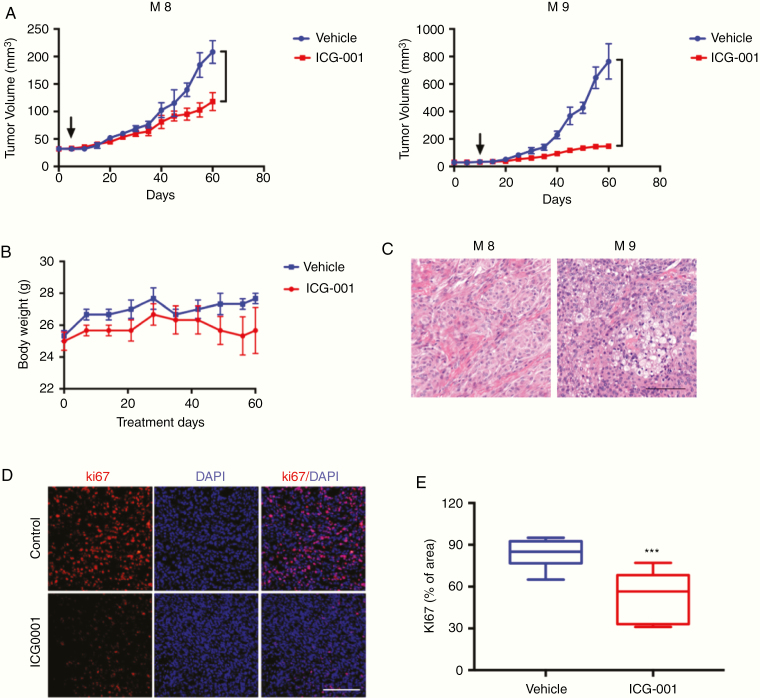
ICG-001 reduces Merlin-negative meningioma patient derived xenografts growth. (A) Patient M8 and M9 xenografts (*NF2*-associated ones) growth are significantly inhibited in mice treated with ICG-001 at 10 mg/kg once a week (i.p.) compared with vehicle-treated groups. (B) The mice did not have significant weight loss within the 8 weeks of treatment. (C-D) H&E staining and Ki-67 histoscore show a reduction in the number of proliferating cells after ICG-001 treatment compared with vehicle-treated groups (bar = 100μm, *P* values are calculated as *t*-Test with Mann–Whitney correction, 5 animals per group).

To validate focused drug screening results in vivo, we tested ICG-001 as a single agent for patients with *NF2*-mutant meningioma (M8 and M9) using PDX models as well as safety considerations measuring by animal weight. ICG-001 significantly inhibited xenografts growth compared with vehicle alone as determined by tumor volume (Fig. 4A). ICG-001 appeared to be nontoxic in vivo, as all of the mice did not have significant weight loss within the 60 days of treatment (Fig. 4B). The in vivo biologic effects of ICG-001 were further addressed through gross and histologic analysis of mice at necropsy (Fig. 4C). The Ki-67 staining assessed on tumors harvested during drug treatment revealed a significant decrease in proliferative activity in ICG-001 versus vehicle control-treated tumors (Fig. 4D and 4E).

### ICG-001 Inhibits Proliferation of Merlin-Negative Meningioma Cells Partly by Attenuating the FOXM1-Mediated Wnt/β-Catenin Signaling

**Fig. 5:**
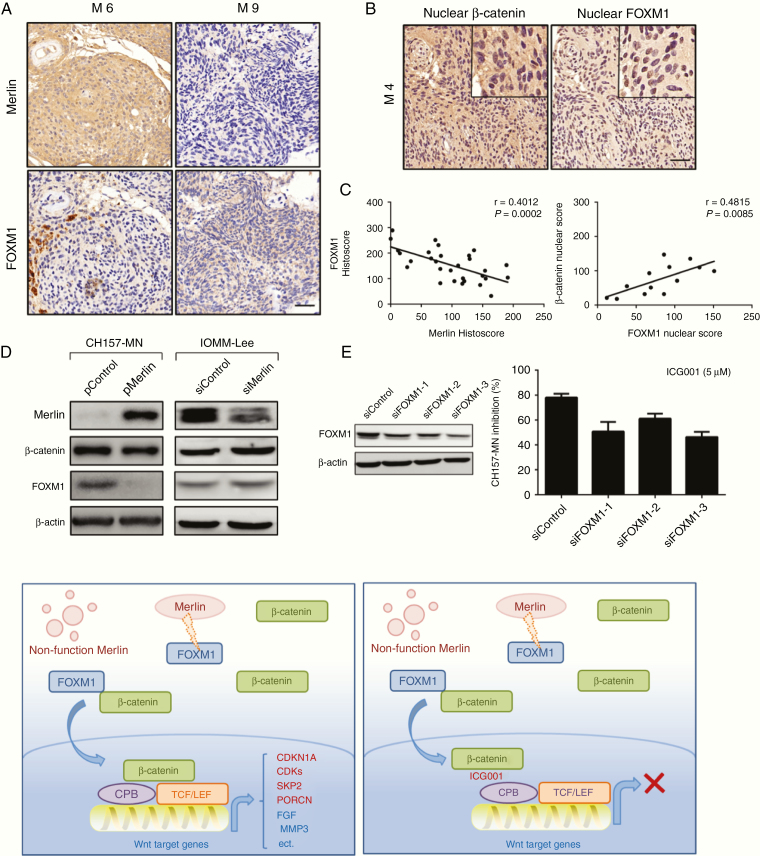
ICG-001 inhibits proliferation of Merlin-negative meningioma cells partly by attenuating the FOXM1-mediated Wnt/β-catenin signaling. (A) Merlin and FOXM1 protein expression in the high-grade meningioma specimens with different background genotype. (B and C) Specimens with nuclear β-catenin staining showed significantly increased nuclear FOXM1 expression. Scale bars 100uM. (D) Increased Merlin expression reduced FOXM1 protein expression, whereas knockdown of Merlin expression led to upregulation of FOXM1 expression. (E) Knockdown FOXM1 in CH157-MN cells resulted in increased sensitivity to ICG001 treatment. (F) Schema Graph: Loss of Merlin stabilizes FOXM1 protein and subsequently enhances the Wnt/β-catenin pathway, resulting in an increased expression of Wnt downstream target genes and promoting meningioma development and progression.

To gain insight into how Merlin loss in meningioma cells dose relate to ICG-001 sensitivity, a further molecular mechanistic investigation was carried out. FOXM1 is a downstream component of the Wnt signaling and is critical for β-catenin transcriptional function in tumor cells.^15,16^ Merlin destabilizes FOXM1 protein, which plays critical roles in aggressive meningiomas.^32^ We thus hypothesized that the Merlin/FOXM1/Wnt signaling axis may play an important role in *NF2*-associated meningiomas. We investigated Merlin and FOXM1 protein expression in a panel of 30 high-grade meningioma samples by IHC staining (Fig. 5A). Overall, a negative correlation between Merlin and FOXM1 expression on TMA specimens was observed (Fig. 5A and 5C). In addition to FOXM1, β-catenin localization was also identified using TMAs. Nuclear β-catenin and nuclear FOXM1 co-expression were present in 43.33% (13 of 30) of all high-grade meningioma specimens. Specimens with nuclear β-catenin staining showed significantly increased nuclear FOXM1 expression (Fig. 5B and 5C).

To further characterize downstream signaling pathways modulated in meningioma cells in response to the Merlin/FOXM1/Wnt signaling axis in vitro, we performed western blots for key signaling proteins with CH157-MN and IOMM-Lee. Clearly, increased Merlin expression reduced FOXM1 protein expression, whereas interference of Merlin expression led to upregulation of FOXM1 expression (Fig. 5D). Increased Merlin expression did not significantly reduce β-catenin protein (Fig. 5D). Finally, interfering FOXM1 in CH157-MN cells resulted in decreased sensitivity to ICG-001 treatment (Fig. 5E), suggesting that ICG-001-mediated growth inhibition of Merlin-negative meningioma cells occurs, at least in part, by attenuating the FOXM1-mediated Wnt/β-catenin signaling.

## Discussion

Over the past decades, our knowledge of meningioma genetics has expended exponentially; ^4,5^ however, the improvement of effective drug treatment has lagged behind.^21–24^*NF2*-mutant meningiomas often cause severe neurologic morbidity and mortality without clearly effective medical treatments.^33,34^ The tumor suppressor *NF2* gene encodes for Merlin protein.^12^ In this study, we first screened Merlin expression in a large cohort through immunohistochemical staining. The results revealed that patients’ outcomes were significantly stratified by Merlin expression on both OS and PFS. Besides, low Merlin expression is associated with MIB-1 labeling index as well as WHO grading according to the WHO 2016 grading criteria for meningioma. Classification of meningiomas as WHO grade I, II, or III is based on several histopathological features, including mitotic activity (number of mitoses per 10 high-powered fields), brain invasion, and/or at least three other aggressive morphologic features.^2^ The degree of mitotic activity is an indicator of the proliferative potential of the tumor. MIB-1 expression in tumor lesions directly correlates with tumors’ proliferation rates and thus may aid in predicting meningioma recurrence.^35^

Based on these results, we look further to find more therapeutic avenues in *NF2*-mutant meningiomas. Specifically, we described 10 high-grade meningioma cases that underwent surgical excision together with focused drug screen using a comprehensive library of up-to-date targeted agents as well as chemotherapeutics. Interestingly, for the *NF2*-mutant cases, ICG-001, a CBP inhibitor of the Wnt/β-catenin signaling pathway, showed the optimal treatment effect both in vitro and PDX models. Merlin acts as a tumor suppressor and is capable of modulating a wide range of signaling pathways, including the Ras/Raf/MEK, PI3K/AKT/mTOR, Rac/PAK/JNK, and Wnt/β-catenin pathways.^9–12^ Numerous studies have documented the amplification or overexpression of positive regulators of the Wnt pathway in meningiomas.^36,37^ Moreover, recent studies have suggested that Merlin can inhibit Wnt/β-catenin signaling through inhibiting β-catenin phosphorylation, thus blocking the translocation of β-catenin from the membrane to the nucleus by inhibiting its dissociation from the adhesion junction.^38^ The fact that Merlin-negative cells were especially sensitive to ICG-001 suggests that the Merlin/Wnt/β-catenin axis may play a role within meningioma tumor cells.

Besides, ICG-001 strongly induced G1 cell-cycle arrest in meningioma cell lines and inhibited the expression of certain genes linked to downstream of the Wnt/β-catenin pathway. Multiple interactions have been described between β-catenin and components of the Wnt enhancer on promoters of Wnt target genes.^39,40^ The central interaction between β-catenin and T cell factor (TCF) is critical to its Wnt activation and represents an attractive target for the general inhibition of canonical Wnt signaling. ICG-001 disrupts the interaction of CBP with β-catenin, specifically downregulating the expression of a subset of β-catenin/TCF-responsive genes.^30,31^*CDKN1A* encodes the cyclin-dependent kinase inhibitor p21 that opposes the function of multiple cyclin-CDK complexes mediating G1 and S cell-cycle progression.^41^ ICG-001 increased CBP occupancy at *CDKN1A* promoter site in tumor cells by increasing the availability of CBP to different subsets of transcription factors mediating the *CDKN1A* expression.^30^ Mutagenesis experiments demonstrated the importance of TCF-binding elements in the *survivin* promoter.^42^ We believe that the observed decrease in survivin expression is also responsible for the induction of apoptosis of the meningioma cells, leading to tumor reduction in vivo. Together, we anticipate that aberrant activation of β-catenin/CBP transcription is a critical event in the development of meningiomas.

FOXM1 is an unexpected but key mediator of the Wnt/β-catenin signaling as it binds to β-catenin, thus enhancing β-catenin nuclear localization and transcriptional activity.^15,16^ Several studies suggested that TCF/LEF and β-catenin coactivators, such as Pygopus/Bcl9, mediate β-catenin nuclear import or retention/anchoring.^43^ In the present study, we systematically analyzed the Merlin expression patterns in meningioma cell lines and tumor specimens. Our results revealed for the first time that the overexpression of FOXM1 attenuated the suppressive effect of Merlin on Wnt/β-catenin signaling, thus sensitizing *NF2*-mutant meningioma cells to ICG-001. To some extent, Merlin may indirectly regulate modification of FOXM1 protein, which decreases the stability of FOXM1.^32^ Although the detailed mechanisms underlying the regulation of FOXM1 protein stability by Merlin require further elucidation, this novel finding regarding Merlin/FOXM1/β-catenin signaling is critical to identifying the molecular mechanisms underlying *NF2*-associated meningioma pathogenesis.

In summary, we have demonstrated for the first time that *NF2*-associated meningiomas are especially sensitive to ICG-001 in vitro and in vivo partly because of the attenuation of the FOXM1-mediated Wnt/β-catenin signaling. Our findings also provide the rationale for the future development of novel mechanism-based therapies for *NF2*-mutant meningiomas.

### Limitations

The present study has several limitations. First, the clinical data set presented here is limited by the selection bias of retrospective nature of this study. Second, about the intratumor heterogeneity of meningiomas, a subset of tumor Merlin-positive tumors might be scored as negative in TMAs. However, this potential misclassification of specimens due to heterogeneity would be considered as randomized distribution, and hence, would most likely have yielded null results. Lastly, the number of targets interrogated with currently available drugs was limited. Additional studies exploring the biology and treatment of *NF2*-mutant meningiomas will certainly be required in the future.

## Supplementary Material

vdz055_suppl_Supplementary_Figure_S1

vdz055_suppl_Supplementary_Figure_S2

vdz055_suppl_Supplementary_Table_S1

vdz055_suppl_Supplementary_Table_S2

vdz055_suppl_Supplementary_Table_S3

vdz055_suppl_Supplementary_Table_S4

vdz055_suppl_Supplementary_Figure_Legend
