# Supplementary figures and images for: The CREB-binding protein inhibitor ICG-001: a promising therapeutic strategy in sporadic meningioma with NF2 mutations
Source: Neurooncol Adv. 2020 Feb 22;2(1):vdz055. doi: 10.1093/noajnl/vdz055 (PMC7212891; doi:10.1093/noajnl/vdz055)

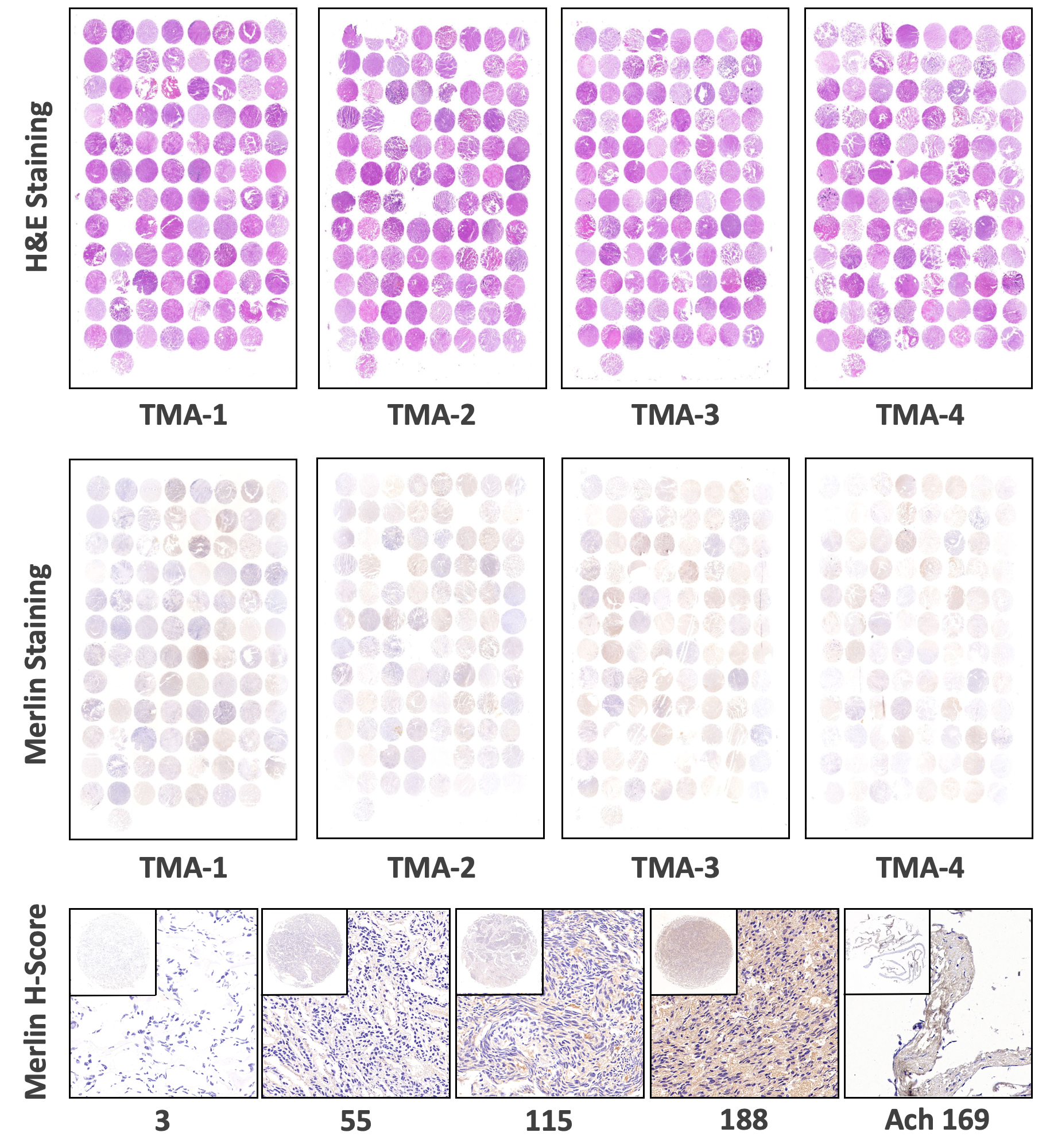

Supplement: vdz055_suppl_Supplementary_Figure_S1 [file vdz055_suppl_supplementary_figure_s1.png]

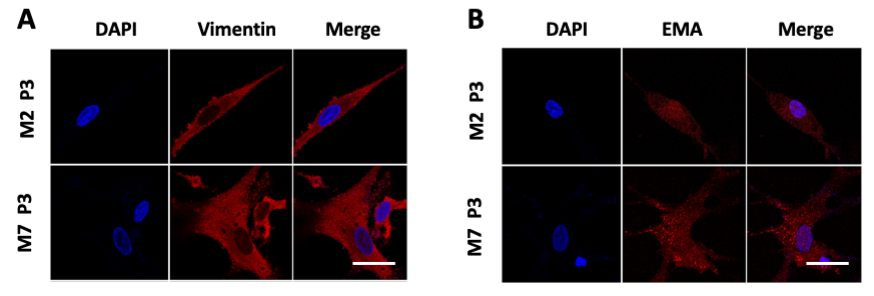

Supplement: vdz055_suppl_Supplementary_Figure_S2 [file vdz055_suppl_supplementary_figure_s2.png]
