# Supplementary material for: The CREB-binding protein inhibitor ICG-001: a promising therapeutic strategy in sporadic meningioma with NF2 mutations
Source: Neurooncol Adv. 2020 Feb 22;2(1):vdz055. doi: 10.1093/noajnl/vdz055 (PMC7212891; doi:10.1093/noajnl/vdz055)
Supplement: vdz055_suppl_Supplementary_Table_S1 [file vdz055_suppl_supplementary_table_s1.docx]

| **Character** |  | **Patients (N=346)** |
| --- | --- | --- |
| **Age (years)** |  |  |
|  | Media | 52.29 ± 15.7 |
|  | Range | 19-86 |
| **Gender, n (%)** |  |  |
|  | Male | 109 (31.5%) |
|  | Female | 237 (68.49%) |
| **Follow up (months)** |  |  |
|  | Media | 76.5 |
|  | Range | 1-142 |
| **Histology at diagnosis, n (%)** |  |  |
|  | Fibrous | 187 (54.05%) |
|  | Meningothelial | 110 (31.79%) |
|  | Atypical | 14 (4.05%) |
|  | Angiomatous | 7 (2.02%) |
|  | Transitional | 6 (1.73%) |
|  | Psammomatous | 6 (1.73%) |
|  | Secretory | 4 (1.16%) |
|  | Microcystic | 3 (0.87%) |
|  | Lymphoplasmacyte-rich | 2 (0.58%) |
|  | Chordoid | 2 (0.58%) |
|  | Clear cell | 2 (0.58%) |
|  | Anaplastic | 2 (0.58%) |
|  | Papilary | 1 (0.29%) |
| **WHO grade, n (%)** |  |  |
|  | Grade I | 325 (93.93%) |
|  | Grade II | 18 (5.2%) |
|  | Grade III | 3 (0.87%) |
| **Extent of resection, n (%)** |  |  |
|  | Simpson grade I | 186 (53.76%) |
|  | Simpson grade II | 124 (35.84%) |
|  | Simpson grade III | 9 (2.6%) |
|  | Simpson grade IV | 27 (7.8%) |
|  | Simpson grade V | 0 (0%) |

**Table S1.** Basic information and clinicopathological characteristics of included patients
