# Supplementary material for: The CREB-binding protein inhibitor ICG-001: a promising therapeutic strategy in sporadic meningioma with NF2 mutations
Source: Neurooncol Adv. 2020 Feb 22;2(1):vdz055. doi: 10.1093/noajnl/vdz055 (PMC7212891; doi:10.1093/noajnl/vdz055)
Supplement: vdz055_suppl_Supplementary_Table_S2 [file vdz055_suppl_supplementary_table_s2.docx]

**Table S2.** Summary of patient-derived meningioma cell lines and corresponding clinical data

| No. | Gender | Age | Histological subtype | WHO Grade | Merlin H-Score | *NF2* mutation | Passage for screening |
| --- | --- | --- | --- | --- | --- | --- | --- |
| M1 | Female | 66y | Atypical | II | 67 | p.Gly165fs | P 3 |
| M2 | Female | 63y | Clear cell | II | 165 | none | P 2 |
| M3 | Male | 42y | Atypical | II | 143 | none | P 3 |
| M4 | Female | 56y | Atypical | II | 92 | p.pro181fs | P 3 |
| M5 | Female | 42y | Atypical | II | 177 | none | P 3 |
| M6 | Male | 22y | Anaplastic | III | 164 | none | P 3 |
| M7 | Female | 65y | Atypical | II | 18 | loss | P 3 |
| M8 | Female | 39y | Atypical | II | 76 | p.Thr59fs | P 3 |
| M9 | Male | 57y | Atypical | II | 32 | loss | P 4 |
| M10 | Male | 61y | Anaplastic | III | 129 | none | P 3 |
| CH157-MN | Female | 41y | N/A | N/A | - | p.V81_splice | / |
| IOMM-Lee | Male | 61y | Anaplastic | III | + | none | / |

**Note.**

y: year.
