# Supplementary material for: The CREB-binding protein inhibitor ICG-001: a promising therapeutic strategy in sporadic meningioma with NF2 mutations
Source: Neurooncol Adv. 2020 Feb 22;2(1):vdz055. doi: 10.1093/noajnl/vdz055 (PMC7212891; doi:10.1093/noajnl/vdz055)
Supplement: vdz055_suppl_Supplementary_Table_S3 [file vdz055_suppl_supplementary_table_s3.docx]

| Inhibitor | Product Name | Target |
| --- | --- | --- |
| E7080 | Lenvatinib | VEGF receptors |
| MI-773 | N/A | MDM2 |
| ICG-001 | N/A | CPB/β-catenine |
| ABT-199 | Venetoclax | Bcl-2 |
| CAL-101, GS-1101 | Idelalisib | p110δ PI3K |
| GDC-0449 | Vismodegib | Hedgehog/Smoothened |
| A1910-5 | N/A | Bromodomain Inhibitor, (+)-JQ1 |
| RAD001 | Everolimus | mTOR |
| GSK2118436 | Dabrafenib | BrafV600 |
| BAY 73-4506 | Sorafenib | Raf-1, B-Raf and VEGFR-2 |
| AZD2281 | Olaparib | PARP1/2 |
| S1267 PLX4032, RG7204 | Vemurafenib | BrafV600E |
| LGK974, HY-17545 | N/A | PORCN |
| GSK-3, A3011 | N/A | Glycogen synthase kinase 3 |
| AGI-6780 | N/A | IDH2/R140Q |
| N/A | Decitabine | DNA methyltransferase |
| AGI-5198 | N/A | IDH1 R132H/R132C mutants |
| 3-deazaneplanocin A (DZNeP) HCl | N/A | S-adenosylhomocysteine hydrolase |
| GSK1120212 | Trametinib | MEK1/2 |
| PD0332991 | Palbociclib | CDK4/6 |

Table S3. Drug list for cell line testing

N/A: not available
