# Supplementary material for: The CREB-binding protein inhibitor ICG-001: a promising therapeutic strategy in sporadic meningioma with NF2 mutations
Source: Neurooncol Adv. 2020 Feb 22;2(1):vdz055. doi: 10.1093/noajnl/vdz055 (PMC7212891; doi:10.1093/noajnl/vdz055)
Supplement: vdz055_suppl_Supplementary_Figure_Legend [file vdz055_suppl_supplementary_figure_legend.docx]

**Supplementary Figure S1.** Immunohistochemistry (IHC) results for the tissue microarray with hematoxylin and eosin (H&E) staining and Merlin staining (n = 355, 2.0x magnification). Representative Histoscore (H-Score) of different Merlin protein expression is shown. Ach: Arachnoidal tissue.

**Supplementary Figure S2.** Characterization of primary high-grade meningioma cells (M2 and M7) with IHC. Single tumor cell with a strong Vimentin (A) and EMA (B) staining. Scale bars 50 uM.
